# When Implementing the Presynch-11/Ovsynch Reproductive Management Program, the Fertility of Lactating Dairy Cows Improved When They Received Timed Artificial Insemination Compared with the Inclusion of Estrus Detection

**DOI:** 10.3390/ani14152235

**Published:** 2024-07-31

**Authors:** Jaimie Marie Strickland, João Paulo Nascimento Martins, Lou Neuder, James R. Pursley

**Affiliations:** 1 Department of Animal Science, Michigan State University, East Lansing, MI 48824, USA; jaimiemstrickland@gmail.com; 2 Large Animal Clinical Sciences, Michigan State University, East Lansing, MI 48824, USA; 3 Department of Medical Sciences, School of Veterinary Medicine, University of Wisconsin-Madison, Madison, WI 53706, USA; jp.martins@wisc.edu

**Keywords:** fertility, Presynch-11-Ovsynch, estrus, pregnancies/AI, lactating dairy cow

## Abstract

**Simple Summary:**

The fertility program Presynch-11-Ovsynch (Presynch-11) improved pregnancies/AI (P/AI) compared with AI following observed standing estrus + timed AI (Estrus + TAI) in first, second, and third+ parities. First parity cows that received AI following an observed standing estrus had greater P/AI compared with second and third+ parity cows. Multiparous cows treated with Presynch-11-Ovsynch had a >60% greater chance of pregnancy compared with cows receiving AI following estrus.

**Abstract:**

Artificial insemination 12 h following observed standing estrus is a standard estimate of the fertility levels of cattle. The main objective of this study was to determine if controlling ovarian development with a fertility program could alter the fertility of lactating dairy cows. Lactating dairy cows (n = 1356) 60–66 days in milk (DIM) were randomly assigned to receive timed AI following Presynch-11/Ovsynch (Presynch-11) or a combination of estrus detection and timed AI using the Presynch-11/Ovsynch program (Estrus + TAI). Cows in standing estrus, following the first two cloprostenol sodium (CLO) injections, in the Estrus + TAI group were artificially inseminated using the AM/PM rule. Cows in this group that were not observed in standing estrus received Ovsynch and TAI beginning 11 d after the second CLO injection. Cows in the Presynch-11 group received two injections of 500 µg CLO 14 d apart but were not observed for estrus. The first GnRH (100 µg) of Ovsynch was administered 11 d following the second CLO injection. All cows in the Presynch-11-Ovsynch group received TAI following Ovsynch. Cows in this treatment were then assigned randomly to receive either CLO or dinoprost 7 d following the first GnRH of Ovsynch. The final GnRH of Ovsynch was administered 56 h later and TAI 16 h after the final GnRH. Pregnancies/AI (P/AI) were greater (*p* < 0.001) for the Presynch-11 group compared with the Estrus + TAI group (45 vs. 31%). Primiparous cows had greater fertility following observed standing estrus compared with multiparous cows. Days in milk at the first AI were greater (*p* < 0.01) in the Presynch-11 group vs. the Estrus + TAI group (98 vs. 80) but less variable (*p* < 0.01). The range of DIM at the first AI was 95 to 101 in the Presynch-11 group and 60 to 101 in the Estrus + TAI group. Within the Presynch-11 group, there were no differences in the rate of luteolysis or P/AI for the prostaglandin type at the final PGF_2α_ of Ovsynch. Multiparous cows treated with Presynch-11 had >60% chance of pregnancy compared with multiparous cows receiving AI following standing estrus. In summary, lactating dairy cows receiving timed AI following the Presynch-11/Ovsynch program had improved fertility compared with a group of cows that received AI following standing estrus or, if not observed in estrus, timed AI. This comparison indicated that controlling ovarian development with GnRH and PGF_2α_ positively impacted the fertility of lactating dairy cows.

## 1. Introduction

Embryonic development of multiparous dairy cows receiving AI following a detected estrus was significantly reduced when compared with nulliparous heifers [1]. This landmark paper prompted significant research to improve pregnancies/AI (P/AI) in multiparous dairy cows [2,3,4]. Currently, synchronization programs that improve the fertility of lactating dairy cows are referred to as ‘fertility programs’, such as Double Ovsynch and G6G [3,5], which pre-synchronize cows to approximately d 5 to 8 of the estrous cycle for the initiation of Ovsynch [6], or Presynch/Ovsynch [7,8], which is dependent upon estrus following each PG to control the time of the estrous cycle at the start of Ovsynch. The first-wave dominant follicle between day 5 and 8 of the estrous cycle has approximately an 85 to 92% chance [5,9] of ovulating following the first GnRH-induced LH surge of Ovsynch and, if so, will initiate a new CL and follicular wave in most cows. This new follicular wave controls the antral age of the ovulatory follicle compared with cows observed in standing estrus. Sartori et al. (2001) [10] demonstrated greater P/AI in cows that ovulated with the first GnRH compared with cows that did not ovulate. Cows that were in the first 4 d of the new follicular wave did not respond to the first GnRH-induced LH surge and ovulate, thus ovulating a follicle with a greater antral age at the final GnRH of Ovsynch [10]. New accessory CL created following GnRH increased the percentage of cows with >2 ng/mL P_4_, at the time of PGF_2α_, by 50% [11]. This may be beneficial to oocyte maturation via a reduction in pulses of LH and FSH during the growth of the ovulatory follicle prior to induced luteolysis. The timing of AI may also benefit fertility programs due to the reduction in variability in cows receiving AI 16 h post LH surges [12]. This allows sufficient time for proper capacitation of sperm prior to or at the time of ovulation. This combination of controlling the antral age of the ovulatory follicle, increased P4 during follicle development, and timing of AI relative to ovulation appears to benefit the fertility of lactating dairy cows [3].

The culling of multiparous dairy cows due to reduced chances of pregnancy results in decreased herd average milk production, increased replacement costs, and ultimately, diminished operational efficiency [13]. Approximately 50% of cows leave the herd before the third lactation [14]. Recent data indicated that ~60% of multiparous cows have a conceptus that attaches to the uterus after the first AI ([15]~d 20–23 post AI) using average fertility service sires. Unfortunately, these cows have a 40% chance of pregnancy loss. Moreover, the likelihood of full-term pregnancies diminishes with each subsequent AI service. Thus, gaining a greater understanding of how to improve the fertility of multiparous cows is critical for dairy farm profitability.

This study aimed to compare two distinctly different reproductive management programs: timed AI only with the fertility program Presynch-11/Ovsynch (Presynch-11) vs. AI following detected estrus following both PGF_2α_ injections within Presynch-11/Ovsynch with timed AI utilized in cows not detected in estrus (Estrus + TAI). We hypothesized that Presynch-11 would improve chances of pregnancy due to its greater control of antral age of the ovulatory follicle, and induction of accessory CL compared with Estrus + TAI.

## 2. Materials and Methods

**Figure 1:**
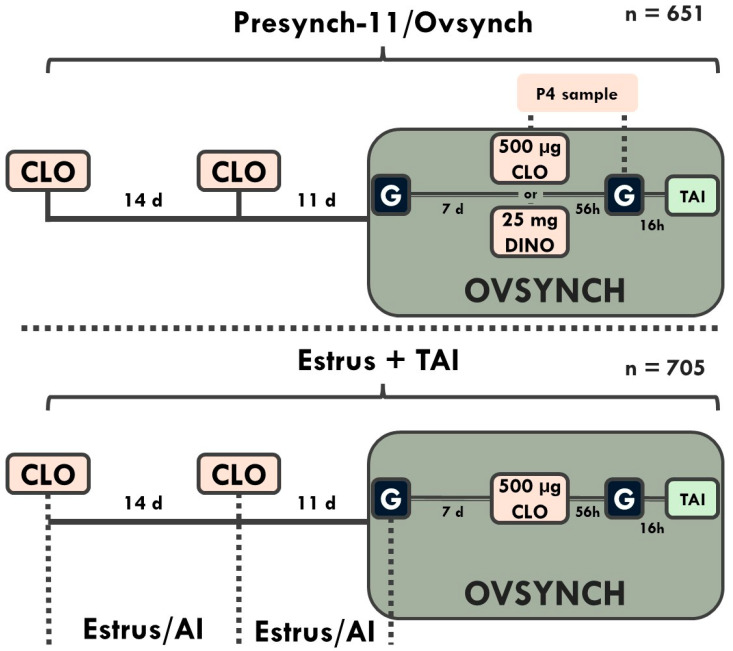
Experimental design to determine the effect of timed AI only with the fertility program Presynch-11/Ovsynch (Presynch-11) vs. AI following detected estrus following both PGF_2α_ injections within Presynch-11/Ovsynch with timed AI (TAI) utilized in cows not detected in estrus (Estrus + TAI). The 1st cloprostenol sodium (CLO) injection in both treatments was initiated between 60 and 66 days in milk (DIM). Cows in the Presynch-11 group all received TAI between 95 and 101 DIM. Estrus detection was performed in the Estrus + TAI group twice daily for the 1st period between 60 and 80 DIM and the 2nd period between 74 and 91 DIM. Cows not observed in standing estrus were treated with Ovsynch 11 days after the 2nd CLO injection and received TAI between 95 and 101 DIM. Cows in the Presynch-11 group were randomly divided into two groups at time of PGF_2α_ of Ovsynch and received either 500 µg of CLO or 25 mg of dinoprost tromethamine (DINO). Blood samples were collected at time of CLO or DINO and at time of final GnRH of Ovsynch for determination of progesterone.

This trial was conducted during the winter months of 2009 at Green Meadow Farms, Elsie, MI. Lactating cows were housed in a free-stall barn with free access to water and were fed a total mixed ration (TMR) three times daily. The TMR consisted of corn and alfalfa silages and corn-soybean meal-based concentrates formulated to meet or exceed the nutrient recommendations for lactating dairy cows [16]. Cows were milked three times daily. Cows in the 1st lactation were grouped separately. Only cows receiving the 1st AI were utilized. All treatments were administered with single-dose syringes in the semimembranosus or semitendinosus muscles of cows by trained personnel from our laboratory. The Institutional Animal Care and Use Committee at Michigan State University approved all procedures. Cows (n = 1356) were randomly assigned by parity to treatment by odd or even ear tag numbers prior to the 60 d voluntary waiting period to receive either Presynch-11 (n = 651) or Estrus + TAI (n = 705; Figure 1).

Cows in Estrus + TAI received intramuscular PGF_2α_ (500 µg of cloprostenol sodium; CLO, Estrumate, Merck Animal Heath, Rahway, NJ, USA) beginning 60–66 days in milk (DIM) and were observed for standing estrus (only cows being mounted) two times daily by farm staff. Cows observed in estrus in the AM received AI in the PM and vice versa. Farm staff routinely walked barns at 12 h intervals to visualize mounting behavior. Estrus + AI received a 2nd injection of 500 µg of CLO 14 d later if they were not already observed in standing estrus. Estrus detection continued for Estrus + TAI for 11 more days. If cows were not observed in standing estrus during this period, they received the 1st GnRH (100 µg of gonadorelin diacetate tetrahydrate; Fertagyl, Merck Animal Health) of Ovsynch; 7 d later, they received PGF_2a_; 56 h later, they received GnRH; and they received TAI 16 h later [6,17]. 

All cows in the Presynch-11 group received TAI following Ovsynch. They were treated with 500 µg of CLO two times, 14 d apart, beginning 60 to 66 DIM. The 1st GnRH of Ovsynch was administered 11 d following the 2nd CLO injection [18]. In addition, cows in the Presynch-11 group were assigned randomly to receive either 500 µg of CLO or 25 mg of dinoprost tromethamine (DINO, Lutalyse; Zoetis, Kalamazoo, MI, USA) at the time of the final PGF_2α_ of Ovsynch. Blood samples were collected at the time of the final PGF_2α_ of Ovsynch and at the final GnRH. Inseminators were not informed if cows were project cows or non-project (2nd + AI) cows when inseminating, so in essence, they were blind to the treatments, even though a greater portion of timed AI cows received AI on Thursdays and Fridays. 

Cows observed with the presence of mucopurulent vaginal discharge or other clinical signs of acute illness at the time of the AI procedure were excluded from the experiment. Four technicians performed AI with commercial semen from multiple sires purchased by the farm. 

All cows were diagnosed for pregnancy 36–42 d following AI unless observed in standing estrus following the 1st AI and re-inseminated. Farm veterinarians blind to the treatments performed pregnancy diagnoses using transrectal ultrasonography (Aloka 500 SSD, Corometrics Medical Systems Inc., Wallingford, CT, USA) 36–42 d following AI.

A subset of cows were considered in the analyses (n = 509) to determine the percentage of cows with complete luteolysis and the effect on P/AI in the Presynch-11 group only. Cows with >2 ng/mL on the day of PGF_2α_ and <0.5 ng/mL 56 h after treatment were considered to have undergone complete luteolysis. Blood samples were collected using Vacutainer tubes without an anticoagulant (BD Vacutainer, Preanalytical Solutions, Franklin Lakes, NJ, USA) and refrigerated for 6 to 12 h. Serum was then separated by centrifugation at 2000× *g* for 20 min at 4 °C and stored at −20 °C for later P4 analyses. Concentrations of serum P4 were quantified with RIA (Coat-A-Count P4, Siemens Diagnostics, Los Angeles, CA, USA). Intra- and inter-assay CVs were 4.9 and 3.2%, respectively. The sensitivity was 0.02 ng/mL.

Binomial variables were analyzed using logistic regression with a generalized linear mixed model implemented with the GLIMMIX procedure of SAS (Version 9.4, SAS Inst., Inc., Cary, NC, USA). The model considered treatment (Presynch-11-Ovsynch vs. Estrus + TAI) and parity (1st, 2nd, or 3rd+) as fixed effects. The 2nd model considered cows with luteolysis in the Presynch-11 treatment vs. cows detected in standing estrus in the Estrus + TAI group. Week was considered a random effect in both models. Two-way interactions of treatment and the parity category were only considered in the model if *p* < 0.20. The other fixed effects considered were estrus (following the 1st and 2nd CLO injections), the Presynch-11 subset with luteolysis, and PGF_2α_ type. Continuous variables were analyzed using a linear mixed model applying the MIXED procedure of SAS for the fixed effects of Presynch-11 vs. Estrus + TAI.

## 3. Results

**Figure 2:**
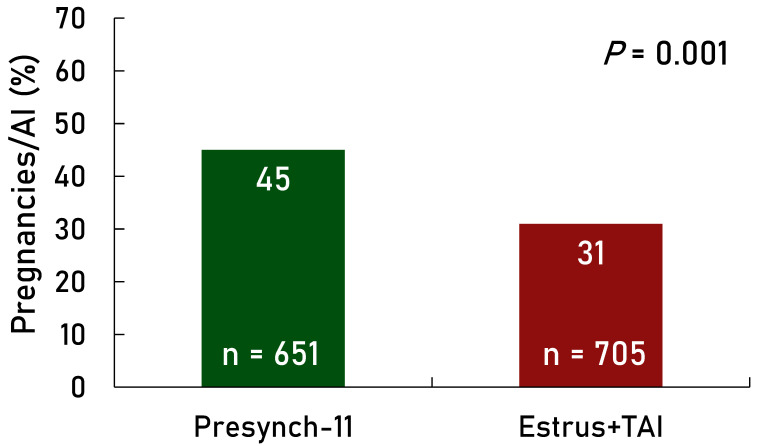
Effect of timed AI only with the fertility program Presynch-11/Ovsynch (Presynch-11; green bar) vs. AI following detected estrus following both PGF_2α_ injections within Presynch-11/Ovsynch with timed AI (TAI) utilized in cows not detected in estrus (Estrus + TAI; maroon bar) on P/AI. Approximately 24% of the Estrus + TAI group were not observed in standing estrus between 60 and 91 DIM and received TAI.

**Figure 3:**
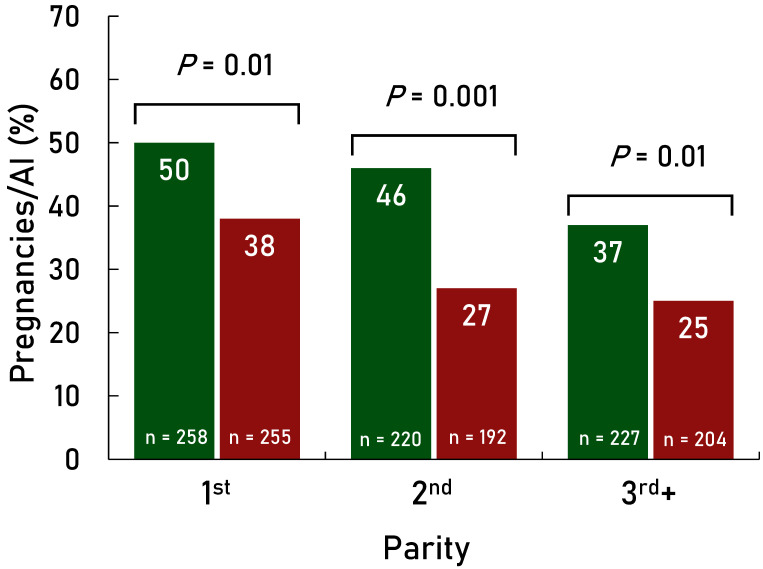
Effect of timed AI only with the fertility program Presynch-11/Ovsynch (Presynch-11; green bars) vs. AI following detected estrus following both PGF_2α_ injections within Presynch-11/Ovsynch with timed AI (TAI) utilized in cows not detected in estrus (Estrus + TAI; maroon bars) on P/AI in first, second, and third + parity lactating dairy cows. Approximately 24% of the Estrus + TAI group were not observed in standing estrus between 60 and 91 DIM and received TAI.

**Figure 4:**
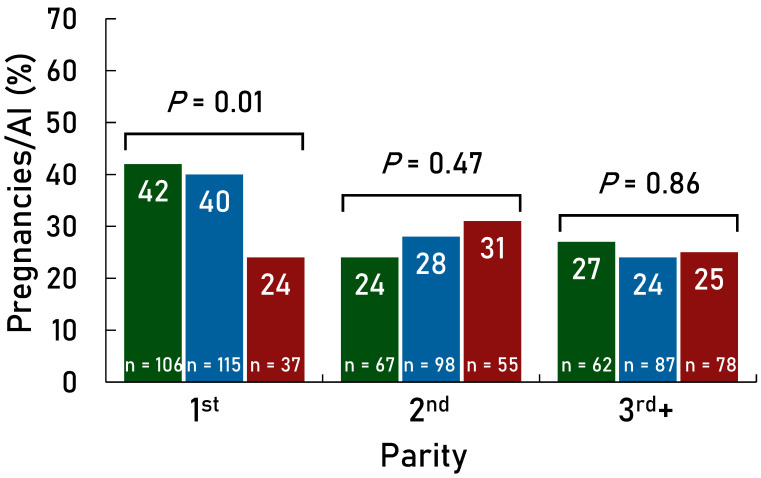
Comparison of P/AI within cows in the Estrus + TAI group for observed standing estrus after first cloprostenol treatment (CLO; green bars) between 60 and 66 days in milk (DIM), after second CLO treatment (blue bars) between 74 and 91 DIM, and cows that were not observed in standing estrus and received TAI following Ovsynch (maroon bars) between 95 and 101 DIM for parities one, two, and three+.

P/AI were greater (*p* < 0.001) for the Presynch-11 group compared with the Estrus + TAI group overall (Figure 2). Also, there was an effect of treatment for each parity group (first, second, and third+) in favor of greater P/AI in the Presynch-11 group vs. the Estrus + TAI group (Figure 3). Days in milk at the first AI were greater (*p* < 0.01) in the Presynch-11 group vs. the Estrus + TAI group (98 vs. 80) but less variable (*p* < 0.01). The range of DIM at the first AI was 95–101 in the Presynch-11 group and 60–101 in the Estrus + TAI group. Primiparous cows in the Estrus + TAI group had greater P/AI compared with multiparous cows (*p* = 0.001; Figure 4) when considering only AI following observed standing estrus after the administration of the first and second CLO injections. Multiparous cows treated with Presynch-11 had a 63% greater chance of pregnancy compared with those treated with Estrus + TAI. 

**Figure 5:**
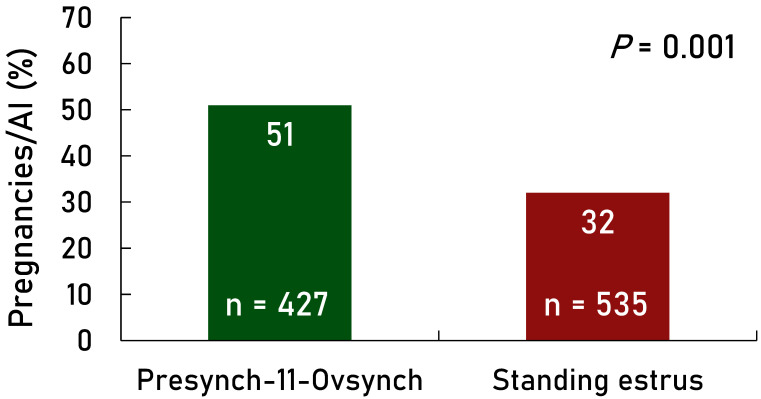
Effect of timed AI only with the fertility program Presynch-11/Ovsynch (Presynch-11; green bar) in cows with luteolysis (<0.5 ng/mL at time of final GnRH of Ovsynch) vs. AI following detected estrus after the first two CLO treatments in the Estrus + TAI group (maroon bar).

Cows in the Presynch-11 group with luteolysis had greater P/AI compared with cows receiving AI following observed standing estrus, following the first and second CLO injections (Figure 5). There was no difference in P/AI for the PGF_2α_ type at the final PGF_2α_ of Ovsynch (45.0% CLO vs. 44% DINO). In addition, there was no effect of the PGF_2α_ type on the percentage of cows with complete luteolysis (<0.5 ng/mL) at the time of the final GnRH of Ovsynch (85% CLO vs. 86% DINO) or parity interaction (*p* > 0.8). Luteolysis, following a single PGF_2α_, for each parity classification, was 87, 85, and 81% for the first, second, and third + parity cows (*p* = 0.12) when the PGF_2α_ types were combined.

## 4. Discussion

It was not clear why P/AI at the first AI were reduced in Estrus + TAI compared with Presynch-11. This discussion is aimed at understanding potential reasons for these differences to improve P/AI in lactating dairy cows.

The range of DIM was greater for the Estrus + TAI group compared with Presynch-11. Approximately three-quarters of the Estrus + TAI group received AI earlier in lactation compared with the treated group. Yet, there were no differences in P/AI (Figure 2) amongst Estrus + TAI cows that received AI from 60 to 80 compared with those 74 to 91 DIM. Also, the added time to the TAI in this group resulted in reduced P/AI, although these could be cows that were not cycling. Thus, there was approximately a 10-day difference in DIM for the Presynch-11-Ovsynch group (95 to 101). It is not clear if this created an advantage for the Presynch-11 treatment; however, this seems unlikely.

Variation in the timing of AI relative to ovulation is clearly greater in cows receiving AI following observed standing estrus compared with those receiving Presynch-11. A hallmark of fertility programs (programs that time the start of Ovsynch during the first follicular wave a few d following deviation) is the specific timing of AI relative to ovulation [19]. In most cases, ovulation occurs ~28 h after the GnRH-induced LH surge in Ovsynch technologies [6]. AI 16 h after the GnRH-induced LH surge provides an ideal period (12 h) for sperm capacitation prior to ovulation. The timing of the LH surge in cows receiving AI following observed standing estrus occurs near time of the first standing event [20]. The timing of ovulation in cows observed for standing estrus is similar (28 ± 5 h) [21] compared with an induced LH surge following the final GnRH of Ovsynch. AM/PM strategies allow for a range in the time of ovulation prior to AI of approximately −20 to 0 h [22]. Based on Pursley et al. (1998), decreases in fertility only occurred when cows received AI after ovulation. So, mistimed AI is an unlikely reason for the difference in P/AI between the treatment groups in this study. Additionally, ovulation failure is an unlikely reason for the difference. There was no difference in ovulation rates in cows that ovulate following estrus vs. timed AI [23].

Primiparous and multiparous cows in the Estrus + TAI group that were not observed for standing estrus and received TAI had significantly less P/AI compared with all cows in the Presynch-11 group. At this point, since 76% of cows in the Estrus + TAI group had received AI following estrus, this was clearly two different groups of cows that likely included a much greater percentage of cows that were anovulatory, infertile, or did not have synchronized ovulation. When we removed cows in the Presynch-11 group that did not have luteolysis and compared the fertility of these cows vs. cows that were observed in standing estrus, there was a 60% increase in fertility in cows with controlled ovarian development (Figure 5). Although the selected group of cows detected in estrus after each CLO treatment in the Estrus + TAI group was assumed to have luteolysis, we did not have P_4_ data on these cows to determine this. It is unlikely that cows in standing estrus would not have luteolysis, considering that low exogenous P_4_ is effective in eliminating standing estrus [24].

The reason for low P/AI in this group may be attributed to how Presynch-11 controls ovarian development. Unfortunately, follicular dynamics were not measured and compared between the two treatments in this study. Potential differences reported in the literature could include the antral age of the ovulatory follicle, the diameter of the ovulatory follicle, the % with double ovulations, and circulating P_4_ concentrations during the growth of the ovulatory follicle [25,26,27].

A perceived advantage of fertility programs is the increase in P_4_ during ovulatory follicle development. This is due to the induction of an accessory CL that adds approximately 50% more P_4_ during the 7 d period of ovulatory follicle development prior to PGF_2α_. Cows that receive PGF_2α_ during a normal estrous cycle likely have less P_4_ on that day, due to it taking place at a random stage of the estrous cycle, compared with a fertility program [2]. But, Cerri et al. (2011) did not observe differences in embryo quality in cows with low vs. high P_4_ during ovulatory follicle development. Also, it did not appear that single ovulatory follicles growing under low P_4_ had a disadvantage in P/AI compared with cows with ovulatory follicles growing under high P_4_ [27]. However, in that study, ovulatory follicle antral age was controlled with GnRH. A greater % of cows with low P_4_ during the growth of the ovulatory follicle had double ovulations, but increased losses due potentially to ipsilateral double ovulations/twinning [27] would have occurred after d 39 pregnancy diagnoses in this study.

Cows receiving AI following observed standing estrus after a complete estrous cycle have a second-wave follicle that would likely have an antral age of ~11 d [25] compared with 9 d in most cows receiving Presynch-11-Ovsynch. Ovulatory follicles with extended antral age have greater chances for reduced oocyte viability [25,28] and embryo quality [29]. Yet, in this study, cows in the Estrus + AI group received CLO to regress CL and control the time to estrus. Cows in this case would more than likely have a shortened antral age of the ovulatory follicle compared with ovulating following a full estrous cycle.

An additional potential explanation for the difference in fertility between the treatments in this study may be due to the interaction of reduced P_4_ during ovulatory follicle development and an extended period of dominance due to the extended time needed for follicular development to induce an LH surge in the estrus group compared with a timed induced LH surge. Thus, the extended time to ovulation in this case is focused more on the later maturation period of the follicle/oocyte. The environment of the oocyte in this type of syndrome may be affected by byproducts of the added time it takes under high LH pulsatility to reach ovulation. These byproducts may include high FSH, maternal age, and the high dry matter intake/high steroid metabolism syndrome in dairy cows. High FSH reduced circulating E_2_ in a super-stimulation model [30], and E_2_ was positively associated with P/AI in dairy cows [5]. Multiparous cows were the primary drivers of reduced P/AI in the Estrus + TAI group in this study. So, in essence, this syndrome may be related only to multiparous cows that are older and produce more milk. Multiparous cows likely have a greater metabolism [26] of E_2_ during the final follicle maturation, leaving less E_2_ in circulation. This, combined with the potential effect of greater numbers of FSH pulses potentially negatively affecting E_2_, in addition to reduced P_4_ prior to luteolysis, may result in an oocyte of reduced competence to develop following fertilization.

The PGF_2α_ product comparison within Presynch-11 indicated no differences in rates of luteolysis or P/AI between the CLO and DINO treatments. This was consistent with previous findings from our laboratory [31,32]. However, progesterone decline during the initial 12 h following injection was greater in the CLO treatment compared with the DINO treatment [32]. The third study from our laboratory indicated that the % of primiparous cows detected in estrus and the overall pregnancy rate was improved with CLO compared with DINO [33].

Improving fertility in cows, in the future, that are being monitored with automated activity monitoring (AAM) is essential to reduce the % of cows culled using these systems. There is a high likelihood that multiparous cows receiving AI following estrus behavior will yield a greater % of cows that become pregnant after 130 DIM. Cows that become pregnant after 130 DIM face increased health issues post parturition and reduced fertility in the next lactation due to excessive body condition losses [34].

In conclusion, timed AI following the Presynch-11/Ovsynch reproductive management program allowed for greater P/AI compared with the detection of estrus following each CLO treatment with TAI in cows not exhibiting standing estrus. Multiparous cows had poor P/AI when receiving AI following a detected estrus, and this was significantly less than the result for cows receiving Presynch-11. There were no differences in P/AI or the percentage of cows with luteolysis between the PGF_2α_ types in the Presynch-11 group. Multiparous cows had a >60% greater chance of pregnancy if ovarian structures were controlled with GnRH and PGF_2α_ compared with AI following observed standing estrus. Optimizing dairy farm profitability hinges on the maintenance of an adequate number of multiparous cows to sustain average daily milk production. These findings underscore the significance of implementing a timed AI fertility program like Presynch-11/Ovsynch for multiparous cows.

## Data Availability

The original contributions presented in the study are included in the paper, further inquiries can be directed to the corresponding author.
